# Efficient binary and QAM optical modulation in ultra-compact MZI structures utilizing indium-tin-oxide

**DOI:** 10.1038/s41598-022-12298-y

**Published:** 2022-05-17

**Authors:** Sohrab Mohammadi-Pouyan, Mehdi Miri, Mohammad Hossein Sheikhi

**Affiliations:** School of Electrical and Computer Engineering, Shiraz University, Shiraz, Iran

**Keywords:** Applied optics, Integrated optics

## Abstract

A design for a CMOS-compatible active waveguide is proposed in which the epsilon-near-zero (ENZ) property of the indium-tin-oxide (ITO) is used to induce large variations in the real and imaginary parts of the waveguide effective index. The proposed waveguide comprises a TiN/HfO_2_/ITO metal–oxide–semiconductor (MOS) structure where the speed and power consumption are significantly improved by the application of the TiN and realization of double accumulation layers in the ITO. Simulations show the insertion loss (IL) of 0.38 dB/μm, extinction ratio (ER) of 11 dB/μm, the energy consumption of 11.87fJ/bit and electrical bandwidth of 280 GHz when the designed waveguide is used as an electro-absorption modulator. The waveguide is then used in an MZI structure to design binary and quadrature-amplitude-modulator (QAM) modulators. For binary modulator, the IL, ER, and V_*π*_L_*π*_ figures of merit are found to be 1.24 dB, 54 dB, and 6.4 V μm, respectively, which show substantial improvement over previous ITO-based designs. In the QAM design, the symmetry in the real and imaginary parts of the waveguide effective index is employed to obviate the need for additional phase shift elements. This considerably reduces the overall length of the proposed QAM modulator and improves efficiency. Simulations show the energy consumption and bit rate, of 2fJ/bit and 560 Gbps, respectively in a 4-QAM modulator with the overall length of 6.2 μm. The symmetry properties of the proposed waveguide can be further exploited to realize quadrature-phase-shift-keying (QPSK) modulators which here is used in combination with the 4-QAM to propose a design for the more advanced modulation scheme of 16-QAM. The design of ITO-based QAM modulators is here reported for the first time and the abovementioned performance parameters show the unique properties of these modulators in terms of footprint, energy consumption and modulation-speed.

## Introduction

CMOS-compatible optical modulators are the principal devices of the silicon-based optical interconnects. Besides compatibility with the CMOS fabrication process flow, these optical modulators should also be compact, high speed, energy-efficient, and have extinction ratio and low insertion loss. All these constraints cannot be completely satisfied in a specific modulator structure and therefore, different platforms have been studied for the realization of optical modulators.

The most important light modulation mechanism in silicon (Si) is the free carrier dispersion (FCD) effect^1^ which has been employed to realize energy-efficient and high-speed optical modulators^2–5^. However, the FCD in Si is weak and to achieve a large extinction ratio the length of the active region should be relatively large^1^. This limits the integration density and increases the overall cost. To resolve this issue, a variety of CMOS-compatible materials such as vanadium dioxide (VO_2_)^6^, barium titanate (BTO)^7^, germanium antimony telluride (GST)^8^, and indium-tin-oxide (ITO)^9–20^, have been studied as the active material of optical modulators. Among these materials, ITO has drawn considerable attention because of its fast and widely tunable FCD effect and epsilon near zero (ENZ) property^10,21^. Electrically induced charge concentration variation in ITO has usually been realized in metal–oxide–semiconductor (MOS) capacitor structures (where the ITO is used as the semiconductor material)^9–17^.

Variation of the ITO carrier concentration changes both the real and imaginary parts of its refractive index which respectively, has been used to design electro-refraction and electro-absorption optical modulators^9–20,22,23^. Even some studies have proposed schemes for complex modulations such a quadrature phase-shift keying (QPSK) to increase the bitrate^10,20^. Application of the voltage induced variations in both real and imaginary parts of the ITO refractive index in an interferometer structure such as Mach–Zehnder interferometer (MZI) or ring resonator; increases the modulator design parameters and can be used to improve its performance. To pursue this idea, we first design an ITO-based active waveguide and adjust its geometrical parameters to increase the variations in both real and imaginary parts of the effective index. The designed active waveguide will be then used as the two arms of an MZI to realize optical modulation. In the active waveguide, a TiN/HfO_2_/ITO/HfO_2_/TiN MOS structure is sandwiched between two Si layers. Application of TiN instead of doped Si (as in^9,11^) as the metallic layer, eliminates the parasitic depletion capacitance of doped Si and increases the overall capacitance of the MOS. This decreases the required bias voltage for inducing an adequate amount of charge in the ITO layer. Noting that the power consumption is linearly related to the capacitance while depends quadratically on the bias voltage (P = 1/2CV^2^), the application of the TiN reduces the power consumption of the waveguide^22^. The previous studies consider the MOS structure as a parallel plate capacitor and assume uniform charge distribution in the ITO layer which is nonrealistic. Here electrostatic simulations are carried out to calculate carrier density of the ITO accumulation layer; *N*_*Acc*_ and the electrical permittivity of the ITO under different bias voltages. The extracted electrical properties of the MOS are then used in Finite-difference-time-domain (FDTD) simulation to calculate the optical properties of the active waveguide.

The optimized waveguide is then used in an MZI structure to realize two-state (binary) and also quadrature amplitude modulation (QAM). The overall length of the proposed binary and QAM modulators are 7.4 μm and 6.2 μm, respectively, and FDTD simulations show the extinction ratio (ER) of 54.04 and insertion loss (IL) of 1.24 for the binary modulator (at the design wavelength of 1.55 μm) while its energy consumption is estimated to be 111fJ/bit. In ITO-based QAM modulators, which to the best of our knowledge had not been reported previously, the simultaneous variations in the real and imaginary parts of the ITO refractive index are employed to improve the modulator performance. For a 4-QAM modulator the IL, energy consumption, and the bit rate are estimated to be 3.9 dB, 2fJ/bit, and 560 Gbps, respectively. A comparison against the previously reported ITO-based modulators shows the superior properties of the proposed modulators in terms of footprint, energy consumption and bit rate.

In what follows we first present the design of the active waveguide. The optical and electrical properties of the designed waveguide will be compared against the properties of the previous designs. The waveguide is then used to design two kinds of MZI based optical modulators; binary and QAM modulators. The characteristics of these modulators are then calculated through simulations and compared against those of the previously reported ITO-based modulators.

## Results

**Figure 1:**
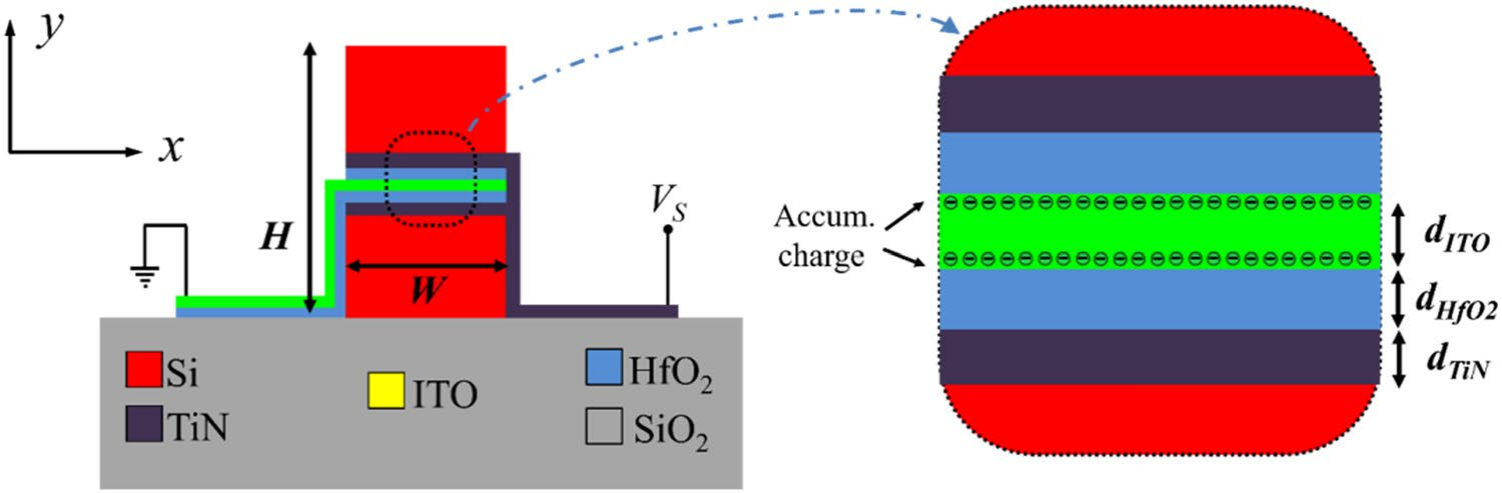
The cross-sectional view of the proposed waveguide with the electrical biasing circuitry. The inset figure shows the charge accumulation in the MOS layers under proper bias condition; *V*_*S*_ > 0.

A cross-section view of the proposed active waveguide is shown in Fig. 1. Compared to the previous vertical MOS waveguides, our waveguide has only one ITO layer and as a result, supports only one interface mode while the presence of more than one interface modes increases the chance of undesired power transfer between optical modes. Also, the application of two-conductor/insulator layers (instead of a single conductor layer used in^9,24,25^) on both sides of the ITO layer enhances the efficiency of the charge accumulation in this layer at the expense of increasing the insertion loss.

Setting *W* and *H* respectively, to 220 nm and 450 nm results in the single transverse magnetic (TM) mode operation in the wavelength range of 1.4 μm < *λ* < 1. The thickness of the HfO_2_ has a less significant effect on the active waveguide properties and is usually set to *d*_*HfO*2_ = 5 nm in previous works^10,24,25^. With this thickness, the HfO_2_ is thick enough to prevent the dielectric breakdown while it is not too thick to decrease the capacitance of the MOS structure and degrade the charge accumulation efficiency. The ITO layer can be considered as a degenerate doped *n*-type semiconductor^26^ and can be formed by tin (Sn) doping of In_2_O_3_ with a concentration of^27^ 10^20^ cm^−3^. TiN, HfO_2_, and ITO layers can be deposited on the Si waveguide by the atomic layer deposition (ALD) process^19,28–30^. The thickness of the ITO and TiN layers (*d*_*ITO*_, and *d*_*TiN*_, respectively) will be calculated in the following subsections. First, electrostatic simulations are carried out to analyze the accumulation process in the ITO and estimate the complex permittivity of this layer by calculating the thickness and carrier density of the accumulation layer (*t*_*Acc*_ and *N*_*Acc*_, respectively). In the last design step, the thickness of the TiN layer is calculated in a way to achieve large variations in the real and imaginary parts of the active waveguide effective and keep the propagation loss at a low level.

In the MOS structure of Fig. 1 applying a positive voltage to the TiN layer while the ITO is grounded; *V*_*S*_ > 0, results in negative charge accumulation in the ITO region and at the ITO/HfO_2_ interfaces. According to the Drude-Lorentz model the change in the ITO carrier concentration demonstrates itself as complex permittivity variations^31,32^:

1 $$\varepsilon \left( \omega \right) = \varepsilon_{\infty } - \frac{{\omega_{p}^{2} }}{{\omega \left( {\omega - i\Gamma } \right)}}\,;\quad \omega_{p}^{2} = \frac{{N_{ACC} q^{2} }}{{\varepsilon_{\infty } m_{n}^{*} }}$$

where *ε*_∞_ = 3.9 is the high-frequency permittivity of ITO, *ω* is the angular frequency, Γ = 1.8 × 10^14^ rad/s is electron relaxation frequency, *m*_n_^*^ = 0.35*m*_0_ is the electron effective mass (*m*_0_ = 9.109 × 10^−31^ kg is the mass of free electrons), *q* = 1.6 × 10^−19^C and *ε*_0_ = 8.85 × 10^−12^ F/m are electron charge and vacuum permittivity, respectively, and *N*_*Acc*_ is the electron concentration in the accumulation layer.

To estimate the complex permittivity of ITO, we should calculate the carrier distribution by numerical solution of the charge continuity equation and Poisson’s equation in the multilayer structure of Fig. 1. The material properties of ITO, TiN, and HfO_2_ used in these simulations are extracted from^33–43^.

**Figure 2:**
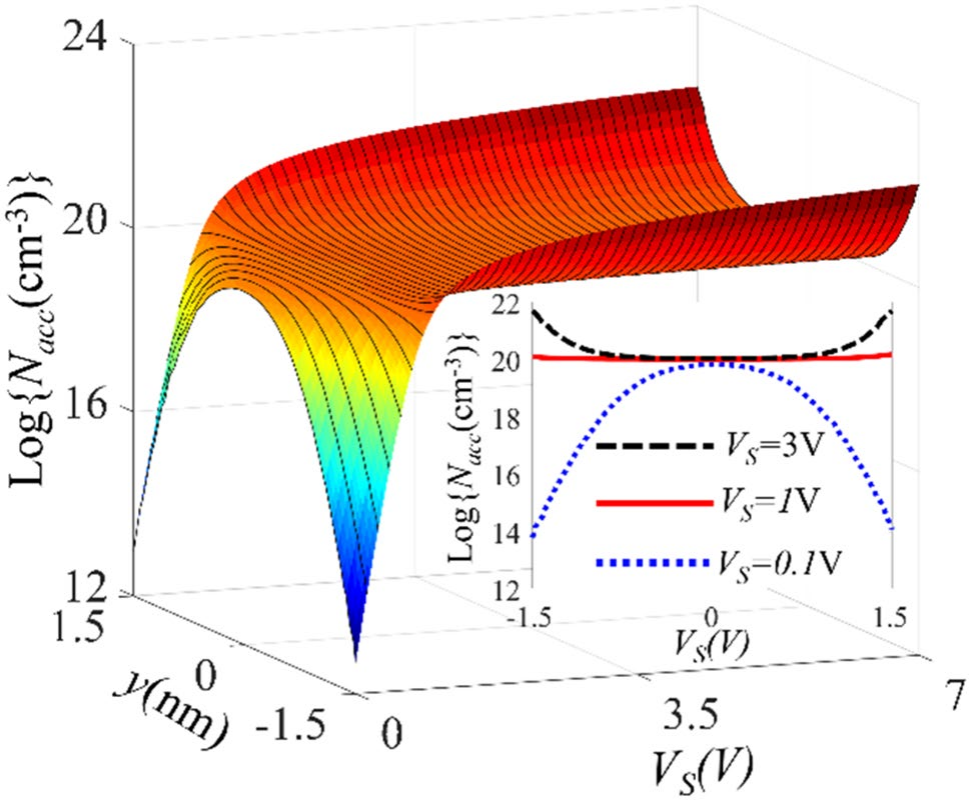
Logarithmic plot of the electron distribution profile in the ITO layer (− 1.5 nm < y < 1.5 nm) as a function of bias voltage; *V*_*S*_.

According to an approximate method based on the Thomas–Fermi screening theory, the thickness of the accumulation layer can be estimated to be^44^ *t*_*Acc*_ ~ 1 nm. Using this approximate value, we set the thickness of the ITO layer to *d*_*ITO*_ = 3 nm in the following electrostatic simulations. Figure 2 shows the electron concentration in the depth of the ITO layer as a function of the bias voltage. As can be seen, for *V*_*S* _< 1 V the electron density in the ITO is smaller than the doing concentration (10^20^ cm^−3^) and for *V*_*S* _= 1 V electron density is equal to the doping concentration everywhere in the ITO layer. This condition is known as the flat-band condition and the corresponding bias voltage is called flat-band voltage; *V*_*FB*_ = 1 V. The electron density reaches its maximum value at the ITO/HfO_2_ interfaces for *V*_*S* _> *V*_*FB*_, and it decays exponentially to the doping concentration of 10^20^ cm^−3^. To show the effect of the bias voltage on the carrier density, the electron density of the ITO layer for V = 0.1 V, 1 V, and 3 V are plotted in the inset of Fig. 2.

**Figure 3:**
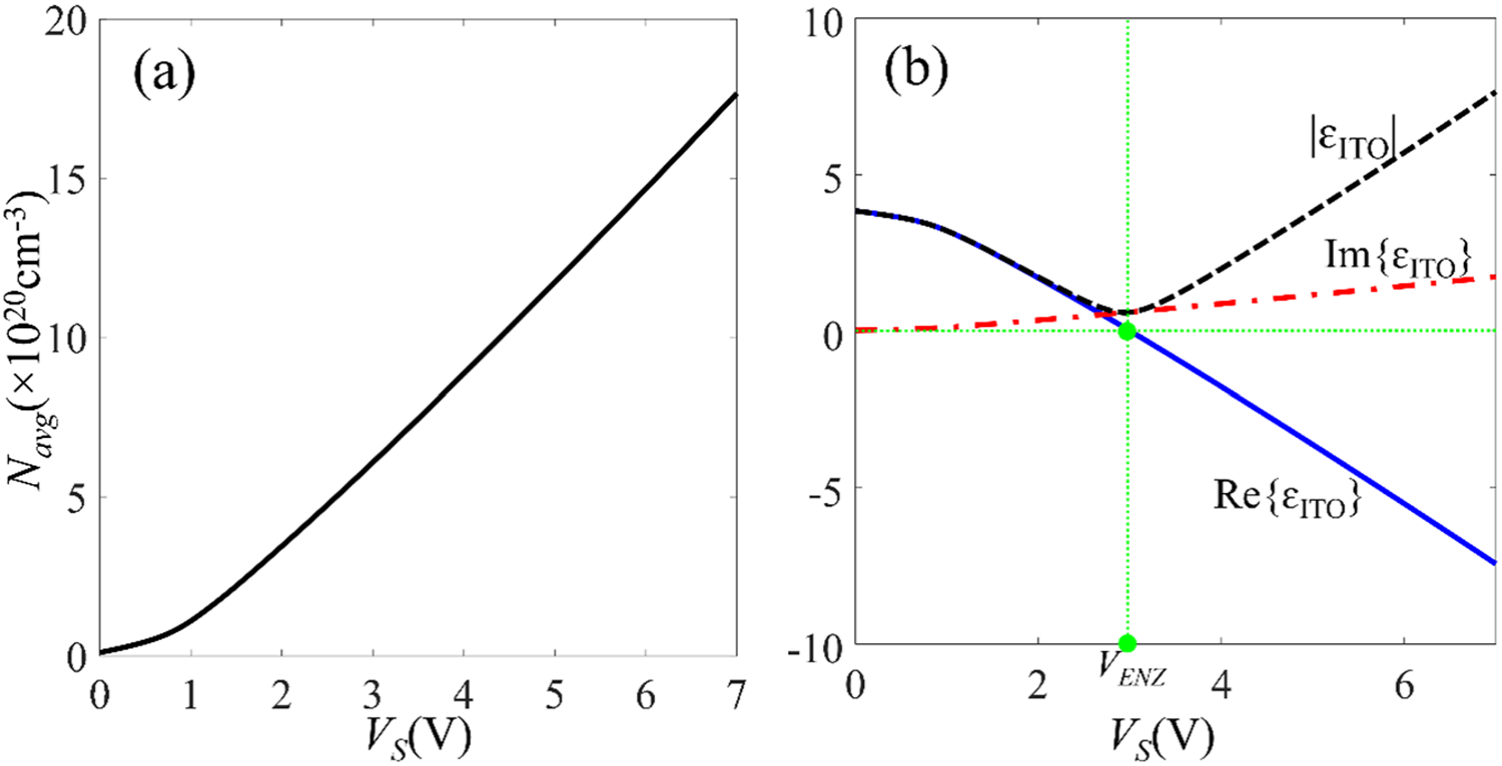
(**a**) Average electron density (*N*_*avg*_) in the ITO layer as a function of the bias voltage *V*_*S*_ and (**b**) complex permittivity of ITO as a function of *V*_*S*_ at the wavelength of 1.55 μm.

According to Fig. 2, for the bias voltage of 1 V < *V*_*S *_< 7 V the thickness of the accumulation layer changes from 1 nm to 1.2 nm therefore, using a 3 nm-thick ITO layer is enough to ensure the formation of two accumulation layers. The average electron density of the accumulation layers (*N*_*avg*_) for different *V*_*S*_ values is plotted in Fig. 3a. For *V*_*S *_> *V*_*FB*_, *N*_*avg*_ increases linearly by increasing the bias voltage with the slope of; *dN*_*avg*_/*dV*_*S* _≈ 2.4 × 10^20^ cm^−3^/V. Therefore, the capacitance of the MOS structure *C*_*eq*_ can be calculated as:

2 $$C_{eq} = \frac{dQ}{{dV_{S} }} = \frac{{d\left[ {WL \times 2t_{ACC} N_{avg} } \right]}}{{dV_{S} }} = \left( {WL \times 2t_{ACC} } \right)\frac{{dN_{avg} }}{{dV_{S} }}$$

where *W* and *L* are the width and length of the active waveguide, respectively. Substituting the geometrical parameters of the waveguide in (2), the unit length capacitance of the structure *C*_*eq*_/*L* is calculated as 18.58 fF/μm.

Using the simulated accumulation charge density in Eq. (1) the real and imaginary parts of the ITO permittivity at *λ* = 1550 nm are also calculated and plotted in Fig. 3b. As *V*_*S*_ increases, the real part of the ITO permittivity decreases till it eventually becomes negative for *V*_*S* _> 3.1 V which can be interpreted as ITO phase transition from semiconductor to metal. The ITO relative permittivity reaches its minimum value of *ε*_*ITO,min*_ = 0.6, at *V*_*S* _≈ 3.1 V and it is corresponding to accumulation density of *N*_*Acc*_ ≈ 6.3 × 10^20^ cm^−3^. This permittivity is smaller than the permittivity of the free space and therefore, the ITO can be considered as an epsilon-near-zero (ENZ) material around this bias voltage^35^ which we denote with *V*_*ENZ*_ = 3.1 V.

Using voltage induced variations in both real and imaginary parts of *ε*_*ITO*_ in an MZI can improve the modulator performance. To do so, we optimize the remaining geometrical parameter of the active waveguide, i.e. *d*_*TiN*_ in way to increase the variations in both real and imaginary parts of *ε*_*ITO*_, when the MOS structure is biased in accumulation regime; *V*_*S*_ > *V*_*FB*_ (waveguide *on*-state), and also keep the propagation loss of the waveguide as low as possible when; *V*_*S*_ ≤ *V*_*FB*_ (waveguide *off*-state).

**Figure 4:**
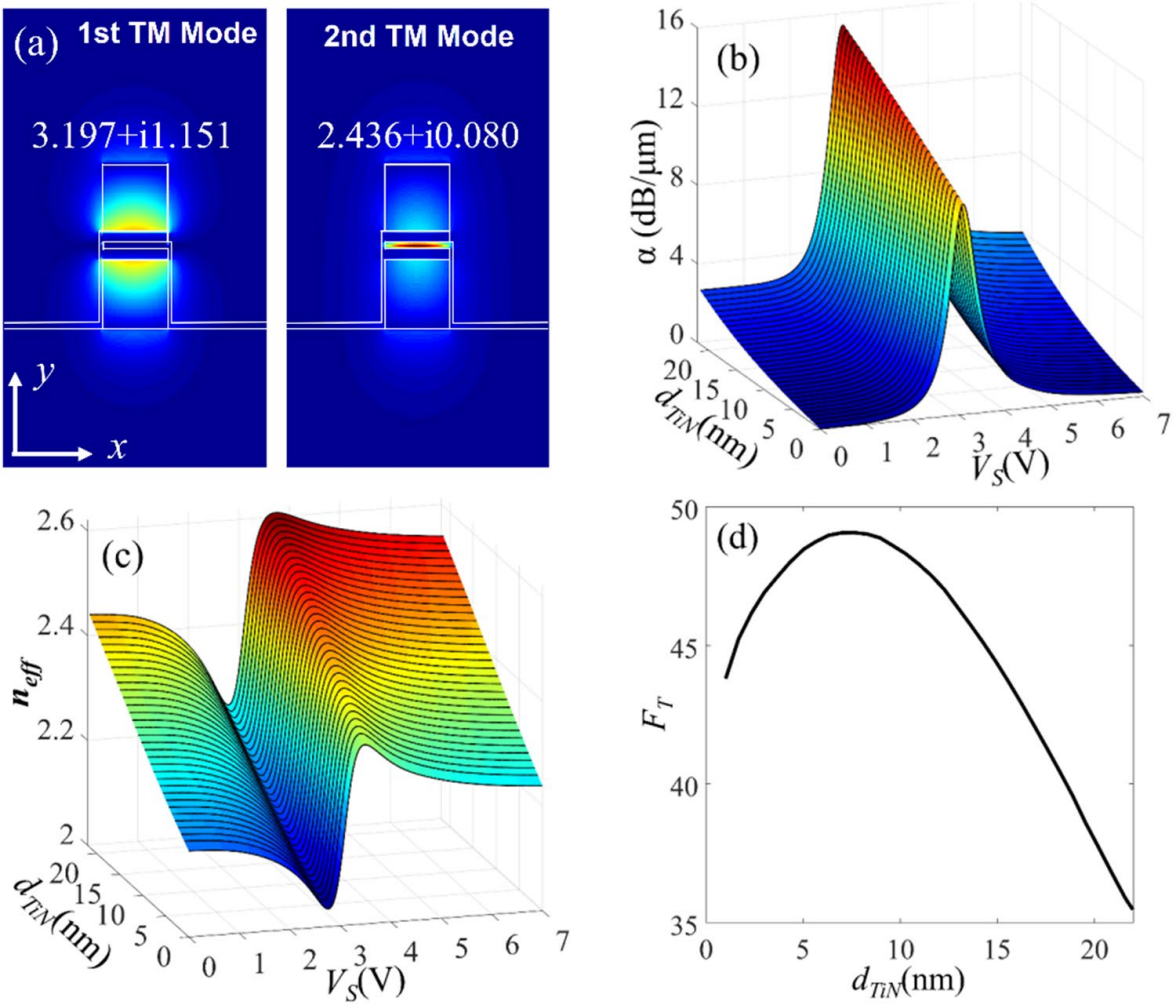
(**a**) Electric field profile of the first (left) and second (right) TM modes of the active waveguide for *d*_*TiN*_ = 25 nm at *λ* = 1550 nm. (**b**) and (**c**), respectively, the variation of the *n*_*eff*_ and *α* of the plasmonic mode of the waveguide for different values of *V*_*S*_ and *d*_*TiN*_. (**d**) Suggested FOM for different *d*_*TiN*_ values.

For *d*_*TiN*_ < 22 nm, the active waveguide supports only one TM and for *d*_*TiN*_ > 22 nm, the waveguide also supports a higher TM mode whose electric field is mainly localized in the Si region and its properties are rarely influenced by the variation of *ε*_*ITO*_. The field profile of these two modes for *d*_*TiN*_ = 25 nm and at *λ* = 1550 nm are presented in Fig. 4a. In the following, we have changed the thickness of TiN in the range of 1 nm < *d*_*TiN*_ < 22 nm, and for each value of *d*_TiN_ calculated the effective index of the waveguide mode for the bias voltage of; 0 V < *V*_*S*_ < 7 V. Figure 4b,c respectively show the real part of effective index; *n*_*eff*_, and the propagation loss; *α*, for different values of the TiN thickness of and bias voltage. From these simulations, the maximum and minimum values of the *n*_*eff*_ and *α* are found for each value of the *d*_*TiN*_ and then used to define the following figure of merit (FOM) for the proposed waveguide;

3 $$FOM\left( {d_{TiN} } \right) = \left( {n_{eff,\max } - n_{eff,\min } } \right) \times \left( {\frac{{\alpha_{\max } - \alpha_{\min } }}{{\alpha_{\min } }}} \right) \times d_{TiN}$$

The first term of (3) evaluates the variations of the real part of the waveguide effective index and *n*_*eff,max*_ and *n*_*eff,min*_ respectively represent the maximum and minimum of *n*_*eff*_ for 0 < *V*_*S*_ < 7 V. The second term evaluates the variation in the propagation loss of the active waveguide and also provides an estimation of the *ER* to *the IL* ratio. This term has been used as the main FOM of ITO-based modulators in some references^10,25^. Here, *α*_*max*_ and *α*_*min*_ respectively represent the maximum and minimum of *α* for 0 < *V*_*S*_ < 7 V. The last term is included to take into account the effect of TiN thickness on the electrical properties of the active waveguide. For example, the electrical 3 dB bandwidth of the active waveguide depends on the resistive-capacitive (RC) time constant of the structure. The equivalent capacitance (*C*_*eq*_) depends on the thickness of the HfO_2_ layer and the carrier density in the ITO layer. The equivalent resistance, on the other hand, is mostly the resistance of the TiN layer and it is inversely proportional to *d*_*TiN*_. Therefore, the thickness of the TiN layer is also included in the evaluation of the waveguide FOM. Using the simulation results of Fig. 4b,c, the FOM of the proposed waveguide is calculated for different values of *d*_*TiN*_ and plotted in Fig. 4d. For *d*_*TiN*_ < 7 nm, increasing the thickness of the TiN layers leads to a slightly better FOM. This can be attributed to the enhancement of the light confinement in the ITO and at the ITO/HfO_2_ interface. At *d*_*TiN*_ = 7 nm, the FOM reaches its maximum value and for *d*_*TiN*_ > 7 nm, thicker TiN layers will be accompanied with larger insertion loss (i.e. larger *α*_*min*_) which degrades the FOM. Therefore, we choose *d*_*TiN*_ = 7 nm as the optimum value for the thickness of the TiN layer. This completes the design steps of the proposed waveguide with the following geometrical dimensions; *H* = 450 nm, *W* = 220 nm, *d*_*ITO*_ = 3 nm, *d*_*HfO2*_ = 5 nm, and *d*_*TiN*_ = 7 nm.

**Figure 5:**
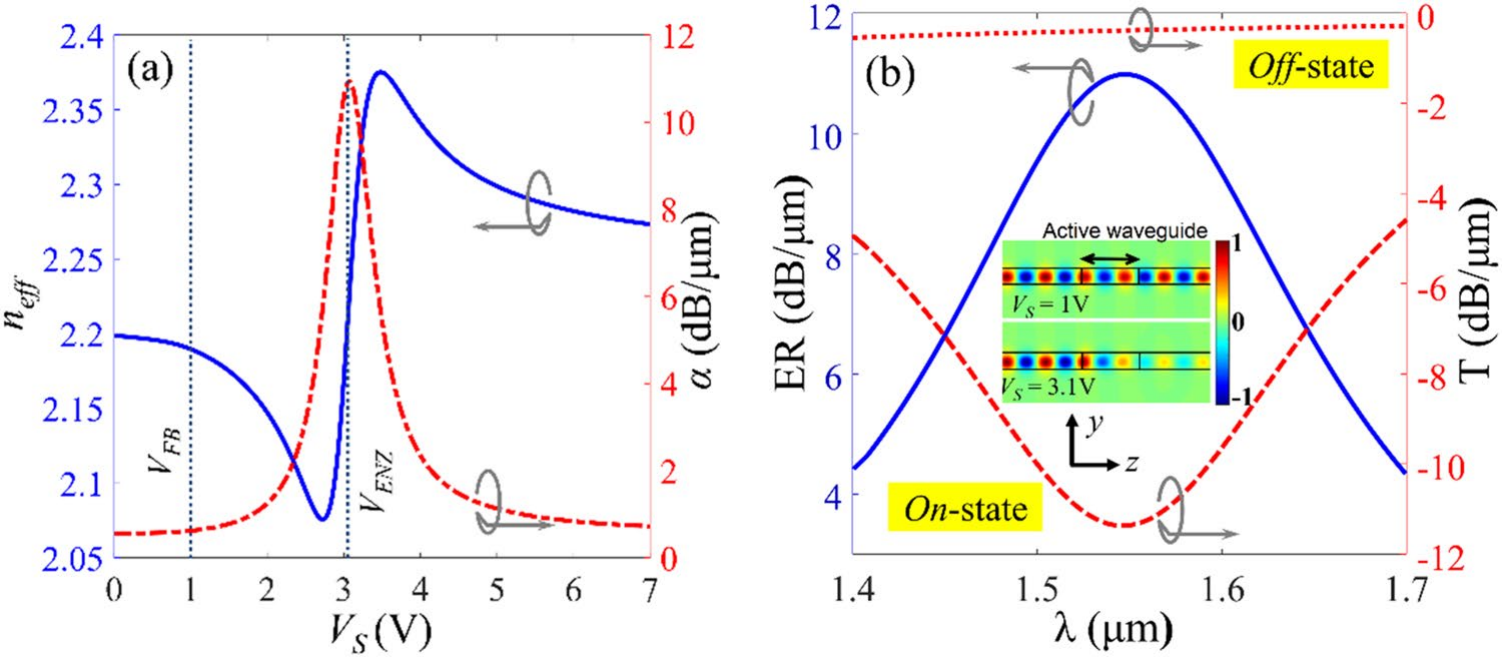
(**a**) The *n*_*eff*_ and *α* of the proposed waveguide as a function of *V*_*S*_ at λ = 1550 nm. (**b**) Power transmission spectrum, IL and ER of the proposed waveguide in *on*- and *off*-states in the wavelength range of 1400-1700 nm. The inset figure shows the electric field distribution in *on*- and *off*-states.

Figure 5a shows *n*_*eff*_ and *α* at the wavelength of 1550 nm for different values of *V*_*S*_. As expected, for *V*_*S *_< *V*_*FB*_ = 1 V, there is no considerable voltage-induced variation in the effective index and the maximum optical loss is obtained for *V*_*S* _= *V*_*ENZ*_ = 3.1 V which are respectively, corresponding to the complex effective indices of *N*_*eff,OFF*_ = 2.1988 + *i*0.0157 and *N*_*eff,ON*_ = 2.2252 + *i*0.3105. To provide an estimation of the electro-optical characteristics of the designed waveguide the transmission spectrum of the 1 μm long active waveguide in the wavelength range of 1400-1700 nm and for the bias voltages, 1 V and 3.1 V are calculated by 3D-FDTD simulation and plotted in Fig. 5b. The *ER* is also calculated and depicted in this figure and the inset shows the electric field profiles for two bias voltages. According to these simulations, at *λ* = 1550 nm the IL and ER of the modulator are 0.38 dB/μm and 11 dB/μm, respectively.

In all FDTD simulations, we have used perfectly matched layer (PML) to truncate the simulation domain in *x*, *y*, and *z* dimensions. Also, to minimize the effect of the absorbing PML layers on the optical modes, these layers are placed at least 2 μm from the end of the active simulation domain. Also, the mesh sizes are refined to minimize the effect of the numerical dispersion. The mesh sizes in *x*, *y*, and *z* dimensions for the strip waveguides are respectively, Δ*x* = 4 nm, Δ*y* = 5 nm, and Δ*z* = 7 nm. In the active waveguide region, on the other hand, very thin layers with very different optical properties are used. Therefore, different mesh sizes should be used in each layer. The *x* and *z* mesh sizes in three layers are set to Δ*x* = 2 nm, and Δ*z* = 5.5 nm, respectively and the *y* meshes for ITO, HfO_2_, and TiN are set to Δ*y* = 0.1 nm, Δ*y* = 0.2 nm, and Δ*y* = 0.25 nm, respectively.

With the applied bias voltage of *V*_*off*_ = *V*_*FB*_ = 1 V and *V*_*on*_ = *V*_*ENZ*_ = 3.1 V, the 1 μm long waveguide of Fig. 5b can be considered as a single bit (*on*–*off*) modulator. The electrical and optical properties of this modulator are compared against those of the previously proposed transparent conductive oxide (TCO)-based *on*–*off* modulators in Table 1. In this table, *L*_*3B*_ represents the required waveguide length for exerting 3 dB loss (when the active waveguide is biased in its high propagation loss), and the electrical bandwidth of the modulator is mentioned as *f*_*3dB*_. Since the FCD process in ITO is very fast the electrical bandwidth of the ITO-based modulators is usually estimated by calculating the RC time delay of the waveguide actuation structure^15^. Using this assumption, the 3 dB bandwidth of the modulator can be approximated as; *f*_*3dB*_ = 1/(2*π R*_*eq*_* C*_*eq*_) where *R*_*eq*_ and *C*_*eq*_ are the equivalent resistance and capacitance of the active waveguide, respectively. The *C*_*eq*_ was previously calculated to be 18.58fF/μm and using the same simulation parameters the equivalent resistance is found to be *R*_*eq*_ = 30Ω μm, which results in *f*_*3dB*_ = 280 GHz. It should be mentioned that the lower resistivity of the proposed waveguide compared to the waveguides of^16,17^, is because of using TiN electrodes instead of heavily doped silicon.

**Table 1:** Comparison of the properties of the proposed waveguide and previous TCO-based *on–off* modulators.

References	IL (dB/μm)	ER (dB/μm)	Energy/bit (fJ)	*f*_3dB_ (GHz)	*L*_*3dB*_ (μm)
Nanowire^12^	0.003	0.11	1300	–	27.28
Hybrid-plasmonic^13^ ^b^	0.04	1	28	300	3
Au slot^14^ ^b^	0.45	2.71	4	100	1.107
Si-rib waveguide^15^	2.6	4.3	22.5	40	0.7
Hybrid-plasmonic^10^	0.03	4.83	14.8	363	0.622
Tri-coupled waveguide^16^	0.003/0.048	0.3/0.33	67.1/22.7	47.5/140	10/9.1
Dual-Polarization^17^ ^a^	0.01	0.2	2115	58.7/65.9	15
Photonic neuron^46^ ^b^	0.2	1	60	25	3
Stub-resonator^47^	2.14	7.14	90.2	78	0.42
Si waveguide^48^ ^b^	2.5	1.62	2100	2.5	1.14
Plasmonic slot-waveguide^49^ ^b^	0.58	2.62	30	250	3
Cadmium oxide^50^	–	5	–	–	1
Photonic memristor^51^ ^b^	0.44	1.2	10^6^	0.03	2.5
This work	0.38	11	11.87	280	0.272

^a^The energy consumption is not reported and it is calculated using the information provided in the paper. The *f*_3dB_ is different for TE and TM polarizations.

^b^These references present measurement results.

The energy consumption of the active waveguide for every bit modulation can be estimated as^22^:

4 $$\frac{Energy}{{bit}} = \frac{{C_{eq} }}{2}\left( {\frac{\Delta V}{{\sqrt 2 }}} \right)^{2}$$

where *ΔV* =|*V*_*on*_* − V*_*off*_ |= 2.1 V is the bias voltage difference between the waveguide *on*- and *off*-states. The energy per bit modulation is calculated to be 20.48fJ/bit.μm. Values reported in Table 1 show that while the energy consumption and the electrical bandwidth of the proposed waveguide are comparable to the best-reported values, it can provide a much higher extinction ratio in the length 1 μm and has much smaller *L*_*3dB*_ length. In the next section, the proposed active waveguide will be used to design MZI based optical modulators. It worth mentioning that, the breakdown electric field of thin-film HfO_2_ is reported to be 12.7MV/cm which^45^ is corresponding to the breakdown voltage of ~ 6.35 V for a 5 nm HfO_2_ film. Since in all proposed devices of this manuscript the applied voltages for switching of the active waveguides are below 4.0 V (i.e. electric field in the HfO_2_ layers will be *E*_*HfO*2_ = 8MV/cm < 12.7MV/cm) and the breakdown of the HfO_2_ will not happen.

Figure 5b shows an interesting property of the proposed waveguide: the real part of the effective index *n*_*eff*_, and propagation loss *α*, are respectively anti-symmetric and symmetric functions of the bias voltage with respect to the *V*_*S*_ = *V*_*ENZ*_ = 3.1 V point. This feature of the proposed waveguide -when combined with the interference properties of photonic elements such as MZI- can be used to design binary, 4-QAM and more advanced modulation schemes such as 16-QAM. In the following subsection, the active waveguide is used to design a binary and a 4-QAM modulator. The optical and electrical properties of these modulators are calculated and compared against the previous ITO-based modulators. Furthermore, a design scheme for a 16-QAM optical modulator is suggested.

### Binary MZI modulator

**Figure 6:**
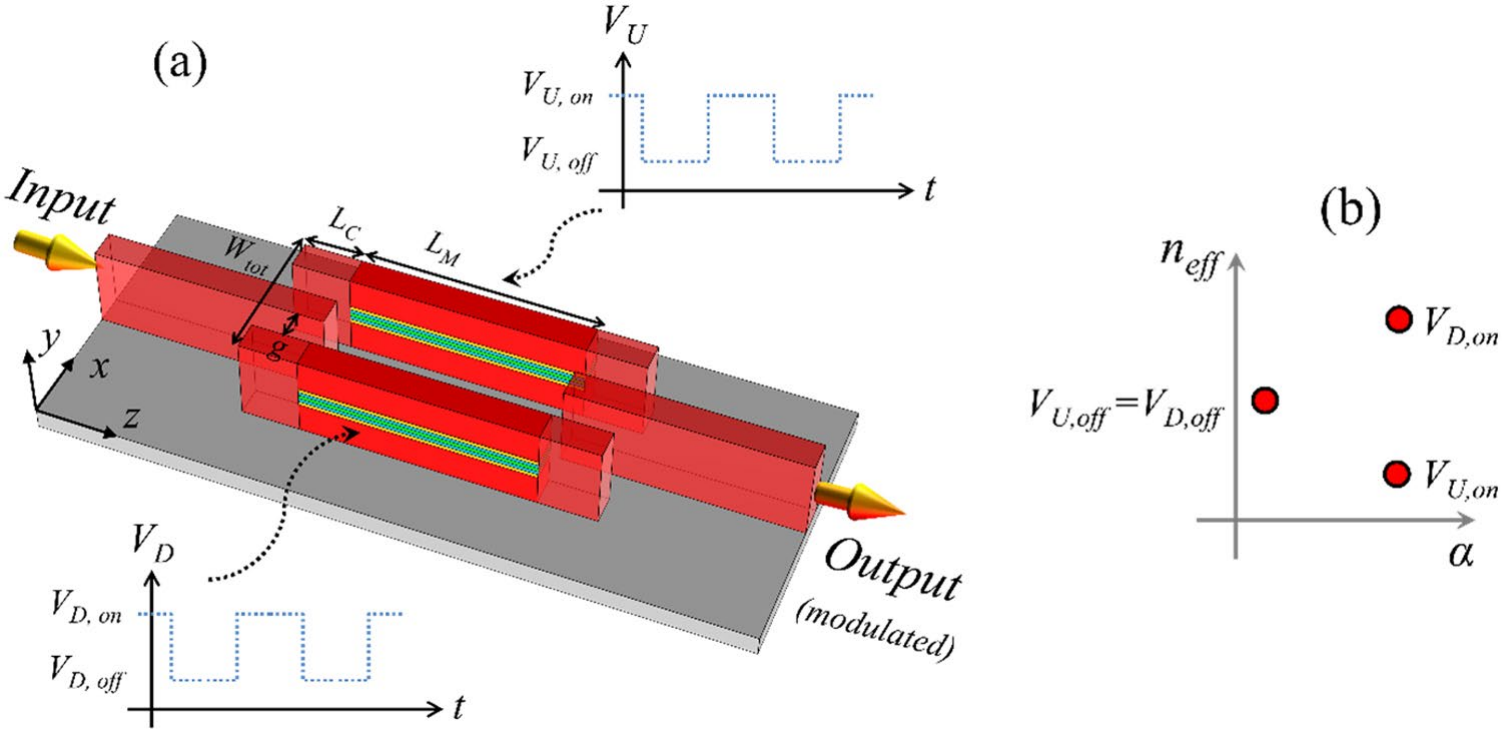
(**a**) The perspective view of the bend-less binary MZI modulator. (**b**) Corresponding effective index and propagation loss of the MZI arms in *on*- and *off*-states.

A perspective view of the binary MZI modulator is presented in Fig. 6a. Because of the symmetry of *n*_*eff*_ and *α* it is possible to choose a pair of bias voltages for upper and lower MZI arms; *V*_*U,on*_ and *V*_*D,on*_ to reach the same propagation loss and different effective index (as shown in Fig. 6b). This way their propagating waves experience different phase shifts and the same loss and the output intensity can be controlled by adjusting the phase shift difference between two arms. The active waveguide bias point is presented on *n*_*eff*_-*α* map in Fig. 6b. At low bias voltage, two active waveguides are in the same point and the required voltage can be any value below flat-band point; *V*_*U,off*_ = *V*_*D,off*_ ≤ *V*_*FB*_. The high bias points on the other hand, should be selected in a way to result in maximum *n*_*eff*_ difference between two waveguides and the same *α*.

Here, instead of Y-junction the MZI is formed by three-waveguide-coupler splitter/combiners to reduce the total footprint of the device. If *E*_*in*_ and *E*_*out*_, respectively represent the input and output electric fields of the MZI, the transmission coefficient of the MZI can be written as^52^:

5 $$T = \left| {\frac{{E_{out} }}{{E_{in} }}} \right|^{2} = \frac{1}{4}\left( {\tau_{1}^{2} + \tau_{2}^{2} + 2\tau_{1} \tau_{2} \cos \Delta \phi } \right)$$

where *τ*_1_ = exp(− *α*_1_*L*_*M*_) and *τ*_2_ = exp(− *α*_2_*L*_*M*_), respectively, are the power transmission of the MZI upper and lower arms, Δ*ϕ* = *ϕ*_1_ − *ϕ*_2_ = (2*π n*_*eff,*1_/*λ*)*L*_*M* _−  (2*π n*_*eff,*2_/*λ*)*L*_*M*_ is the phase difference between two arms, *α*_*i*_, and *n*_*eff,i*_ (*i* = 1, 2) are the propagation loss and the effective index of active waveguides, respectively, and *L*_*M*_ is the length of active waveguides.

To maximize the ER of the modulator, the propagation loss of two arms should be the same; *α*_1_ = *α*_2_ and Δ*ϕ* should be equal to “*π*” and “0” in *on*- and *off*-states, respectively. Also, to minimize the total length of the device and reduce its equivalent capacitor and energy consumption, the bias points should be selected in a way to maximize; Δ*n*_*eff*_ = *n*_*eff*,1_ − *n*_*eff*,2_. According to Fig. 5b, setting the bias voltages to; *V*_*U,off*_ = *V*_*U,off*_ = *V*_*FB*_ = 1 V, *V*_*U,on*_ = 2.8 V and *V*_*D,on*_ = 3.37 V, result in *Δn*_*eff*_ = 0.2886 and *α*_1_ = *α*_2_ = 7.09 dB/μm. This is equivalent to the active waveguide length of; *L*_*M*_ = (2*λ*/Δ*n*_*eff*_) = 2.7 μm at *λ* = 1.55 μm.

**Figure 7:**
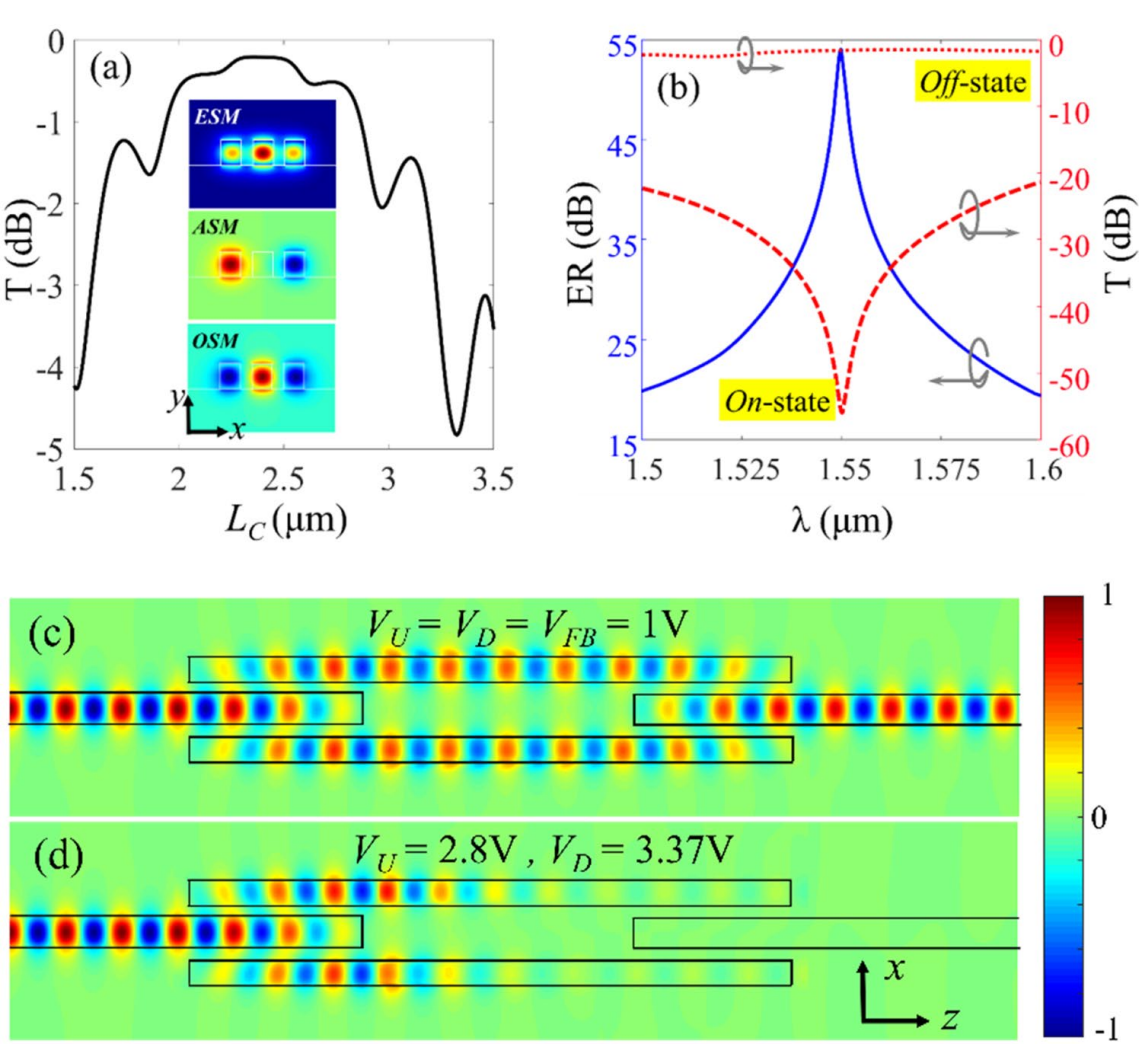
(**a**) The power transmission of the splitter/combiners as a function of *L*_*C*_. The inset figure shows the splitter/combiner super modes at λ = 1.55 μm. (**b**) The transmission spectrum of the MZI modulator in *on*- and *off*-states and its ER in the wavelength range of 1.5 μm < λ < 1.6 μm. (**c**) and (**d**) Electric field distribution in the modulator in *off*- and *on*-states at λ = 1.55 μm, respectively.

The coupling region of the three-waveguide-coupler supports three super-modes; even-symmetric super-mode (ESM), antisymmetric super-mode (ASM), and odd-symmetric super-mode (OSM) whose electric field profiles are shown in the inset of Fig. 7a. We use the coupled-mode theory (CMT) to find the approximate length of the coupling region *L*_*C*_ and then use FDTD simulations to fine-tune this value for optimum coupling. We denote the effective indices of the abovementioned super-modes with *n*_*ESM*_, *n*_*ASM*_, and *n*_*OSM*_, respectively. For efficient power transfer the effective index of the super modes should satisfy the condition of^53^; *n*_*ASM*_ = (*n*_*ESM*_ + *n*_*OSM*_)/2. The effective indices of the supper modes can be adjusted by changing the coupling gap between the waveguides; *g* (see Fig. 6a). According to our calculations, for the coupling gap of *g* = 170 nm the super mode effective indices are *n*_*ESM*_ = 2.408, *n*_*ASM*_ = 2.245, and *n*_*OSM*_ = 2.082, which satisfy the required condition. Therefore, the coupling gap is set to 170 nm and the overall width of the MZI structure will be *W*_*tot*_ = 1 μm.

Based on the CMT formulation the length of the coupling region (at the design wavelength of λ) can be calculated as^53^:

6 $$L_{C} = \frac{\lambda }{{2\left( {n_{ESM} - n_{OSM} } \right)}}$$

which results in *L*_*C*_ = 2.37 μm for the design wavelength of *λ* = 1.55 μm.

To verify this result and also evaluate the coupling loss, we changed the length of the coupling region in the range of 1.5 μm < *L*_*C*_ < 3.5 μm and calculated the total optical transmitted power at *λ* = 1.55 μm (using 3D-FDTD). The results presented in Fig. 7a, shows that the maximum power transfer can be achieved for *L*_*C* _= 2.35 μm that is corresponding to the coupling loss of 0.2 dB. Considering the active waveguide length of *L*_*M*_ = 2.7 μm the overall length of the MZI modulator will be (2*L*_*C*_ + *L*_*M*_) = 7.4 μm. Using these dimensions, we have simulated the MZI modulator and calculated the power transmission for the *on*- and *off*-states for the wavelength range of 1.5 μm < λ < 1.6 μm. Figure 7b shows the transmitted power in *on*- and *off*-states and also the ER of the modulator. At the wavelength of λ = 1.55 μm, the ER and IL of the modulator respectively, are calculated to be 54.04 and 1.24 dB and in the wavelength range of 1.5–1.6 μm and the modulator ER is above 20 dB and its IL is lower than 1.35 dB. Electric field distribution in the MZI modulator in *off*- and *on*-states are also depicted in Fig. 7c,d, respectively.

An important FOM of the MZI based binary modulator is the product of the half-wave voltage and the length of the modulator’s active waveguide segments; *V*_*π*_*L*_*π*_. For the proposed push–pull MZI modulator the FOM can be calculated as:

7 $$V_{\pi } L_{\pi } = \max \left\{ {\Delta V_{1} ,\Delta V_{2} } \right\}L_{M}$$

where Δ*V*_1_ and Δ*V*_2_ is the difference between the *on* and *off* bias voltages of the modulator arms. Using; Δ*V*_1_ = *V*_*U,on*_ − *V*_*U,off*_ = 1.8 V, Δ*V*_2_ = *V*_*D,on*_ − *V*_*D,off*_ = 2.37 V, and *L*_*M* _= 2.7 μm in the above formula, *V*_*π*_*L*_*π*_ is calculated to be 6.4 V μm. Furthermore, the energy consumption for every bit modulation can be estimated as^19^:

8 $$\frac{Energy}{{bit}} = \frac{{C_{arm} }}{4}\left( {\Delta V_{1}^{2} + \Delta V_{2}^{2} } \right)$$

where *C*_*arm*_ = *C*_*eq*_ × *L*_*M*_ = 50.16fF is the equivalent capacitor of each MZI arm. Equation (10) results in the energy consumption of 111fJ/Bit. A comparison between the parameters of the proposed modulator and some of the most compact MZI-based modulators is provided in Table 2.

**Table 2:** Comparison of proposed binary modulator performance and previous compact MZI based modulators.

Device type	Active length (μm)	IL (dB)	Energy (fJ/bit)	V_π_L_π_ (V μm)	Overall footprint^a^ (μm)^2^	Bit rate	Optical bandwidth (nm)
ITO based^19^ ^c^	32	~ 6	11,000	520	> 32	–	–
All plasmonic^48^ ^c^	10	7–9	25	60	~ 24	1.1 THz	100
Photonic Crystal based^49^ ^c^	5.78	1.08	6000	5.78	> 13.82	~ THz	3–5
This work	2.7	1.24	111	6.4	7.4	280 GHz	> 100^b^

^a^Required electrical contacts are not considered in the estimation of the overall footprint.

^b^Here optical bandwidth refers to the wavelength range where the ER of the proposed modulator is above 20 dB and its IL is below 1.4 dB.

^c^These references present measurement (experimental) results.

Compared to the previous MZI-based modulators the proposed modulator consumes relatively low energy, has very low IL and *V*_*π*_*L*_*π*_, and has the smallest active waveguide length and overall footprint. The very low *V*_*π*_* L*_*π*_ FOM, active length and footprint of the proposed MZI modulator can be attributed to the fact that the MOS structures are biased around the ENZ point where very large variations the waveguide effective index can be achieved with relatively small changes in the bias voltage. The optical bandwidth of the modulator is also ultra-wide and is limited by the splitter/combiner’s spectral response.

In what follows we will use large variations in both effective index and propagation loss of the MOS active waveguide to design an ultra-compact and high-speed 4-QAM optical modulator.

### QAM modulator with integrated phase shifter

**Figure 8:**
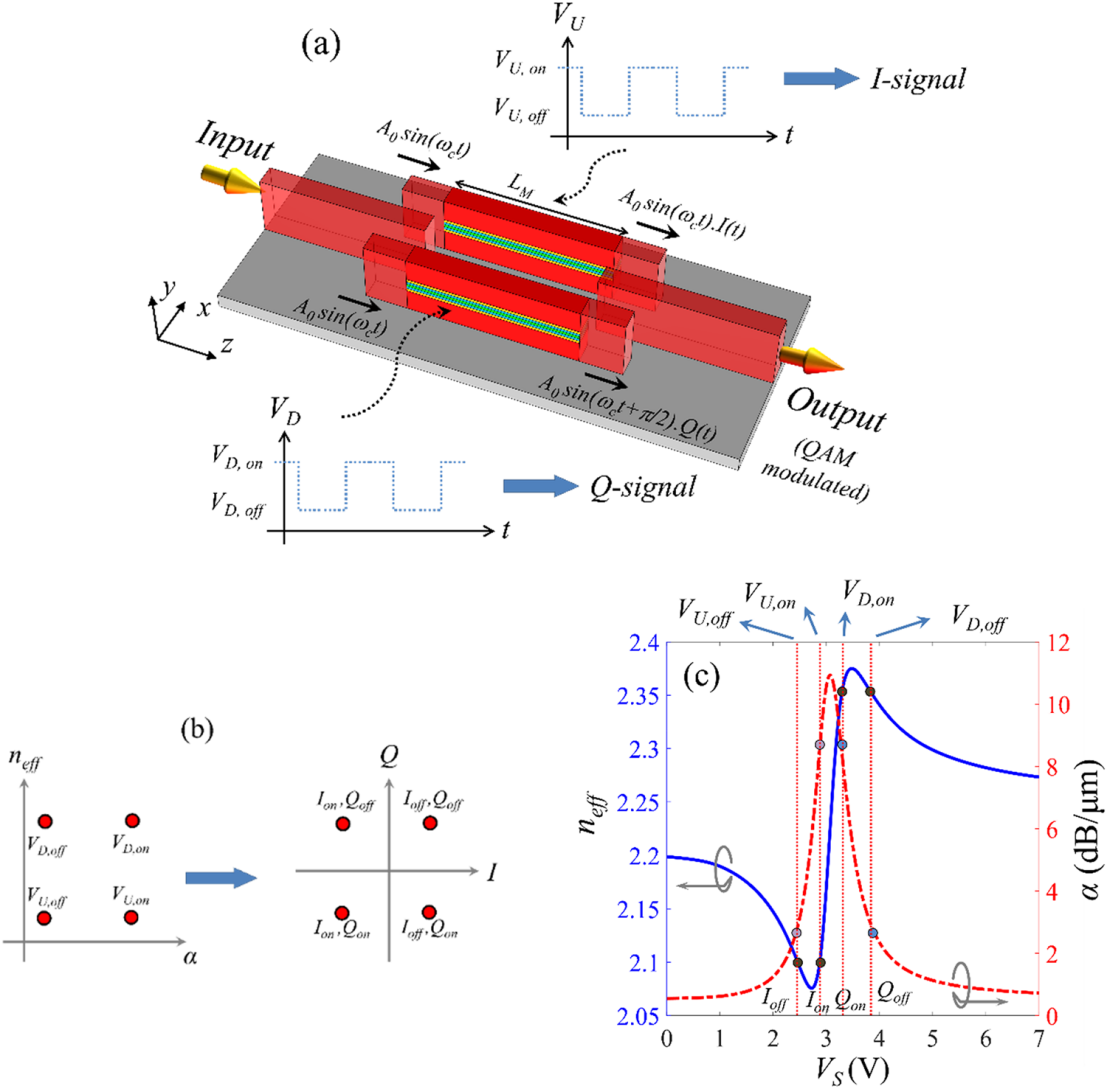
(**a**) schematic view of the proposed QAM modulator with its signaling configuration. (**b**) Bias points of the active waveguides in MZI arms in *n*_*eff*_-*α* map (left), and the constellation points of the QAM modulator in *I*–*Q* plane (right). Each point is denoted by states of the *I*- and *Q*-modulators. (**c**) Selection scheme for the bias points of the MZI arms on the *n*_*eff*_-*α* map.

A QAM modulator comprises two distinct amplitude modulators with the same carrier frequency (*ω*_*c*_ in Fig. 8a) where the carrier wave of one modulator experiences a phase shift of *π*/2 with respect to the other. The output QAM modulated signal is formed by combining these two orthogonal waves. Optical QAM modulation can be realized by an MZI structure with two active waveguides in its arms and an integrated *π*/2-phase shifter in one arm^50,54–57^. The modulating signal of the arm with phase shifter is called *Quadrature signal* (*Q*-signal) and the other is called *In-phase signal* (*I*-signal). The required *π*/2-phase shift can be realized through a delay line which significantly increases the length and overall footprint of the modulator. Instead, we can use the anti-symmetric behavior of the active waveguide effective index to the bias voltage variations (around *V*_*S*_ = *V*_*ENZ*_ point) and realize the phase shift by selecting proper bias point for the MZI arms. It should be mentioned that ITO-based QAM modulators have not been proposed previously. Also, as is going to be shown, elimination of the delay line substantially reduces the length of the proposed QAM modulators compared to the previously reported optical QAMs.

A perspective view of the proposed 4-QAM modulator along with the *I*- and *Q*-modulating signals, and the formula for the input/output signals of each MZI arm is shown in Fig. 8a. The geometrical parameters of the active waveguide and the three-waveguide-couplers are the same as the binary MZI modulator, and the QAM modulator design parameters include the length of the MZI arms; *L*_*M*_ and the bias points of these waveguides; *V*_*D,on*_, *V*_*D,off*_, *V*_*U,on*_, and *V*_*U,off*_. To produce the phase shift of *π*/2 between the MZI arms (see Fig. 8a), the two arms should have different *n*_*eff*_ values. Also, *n*_*eff*_ of each arm should remain the same for both bias voltage levels while its propagation loss varies. Furthermore, to balance the QAM constellation points, the amplitude of the output signals of the two MZI arms should be the same in both *on* and *off* modulation states. In other words, as depicted in Fig. 8b the bias point of the MZI arms should be located at the vertices of a square in *n*_*eff*_ − *α* map or equivalently the modulating signals should be located on the same pattern in *I − Q* constellation map. According to our calculations, the minimum active waveguide length can be achieved by setting the bias points of the upper and lower MZI arms to; (*V*_*U,off*_, *V*_*U,on*_) = (2.44 V, 2.91 V), and (*V*_*D,on*_, *V*_*D,off*_) = (3.26 V, 3.85 V), respectively. The bias point of the MZI arms and their corresponding *n*_*eff*_, and *α* values are shown in Fig. 8c. With these bias voltages the (*n*_*eff*_, *α*) values of the upper MZI arm in *on*- and *off*-states will be (2.1, 8.47 dB/μm) and (2.1, 2.6 dB/μm), respectively and the values for the lower arm in *on*- and *off*-states will be (2.36, 8.47 dB/μm) and (2.36, 2.6 dB/μm), respectively. The above values result in the effective index difference of Δ*n*_*eff*_ = 0.26 between two arms and therefore, the required active waveguide length for the *π*/2 phase difference is *L*_*M*_ = 1.5 μm (at the wavelength of 1.55 μm). Adding the length of the three-waveguide couplers results in the overall modulator length of 6.2 μm. The optical power transmission of the structure in *off*- and *on*-states at *λ* = 1.55 μm are calculated to be − 3.9 dB and − 12.7 dB, respectively and energy consumption per bit is estimated to be ~ 2fJ/bit. This value is estimated by dividing the energy consumption for every state change for an active waveguide length of *L*_*M*_ by the number bits per symbol^55^. Also, because of the QAM operation, the electro-optical bandwidth of the device can be calculated by multiplying the speed of active waveguide *n*_*eff*_-*a* state change by the corresponding number of bit for every state change; 280 GHz × 2 = 560Gbit/sec.

**Figure 9:**
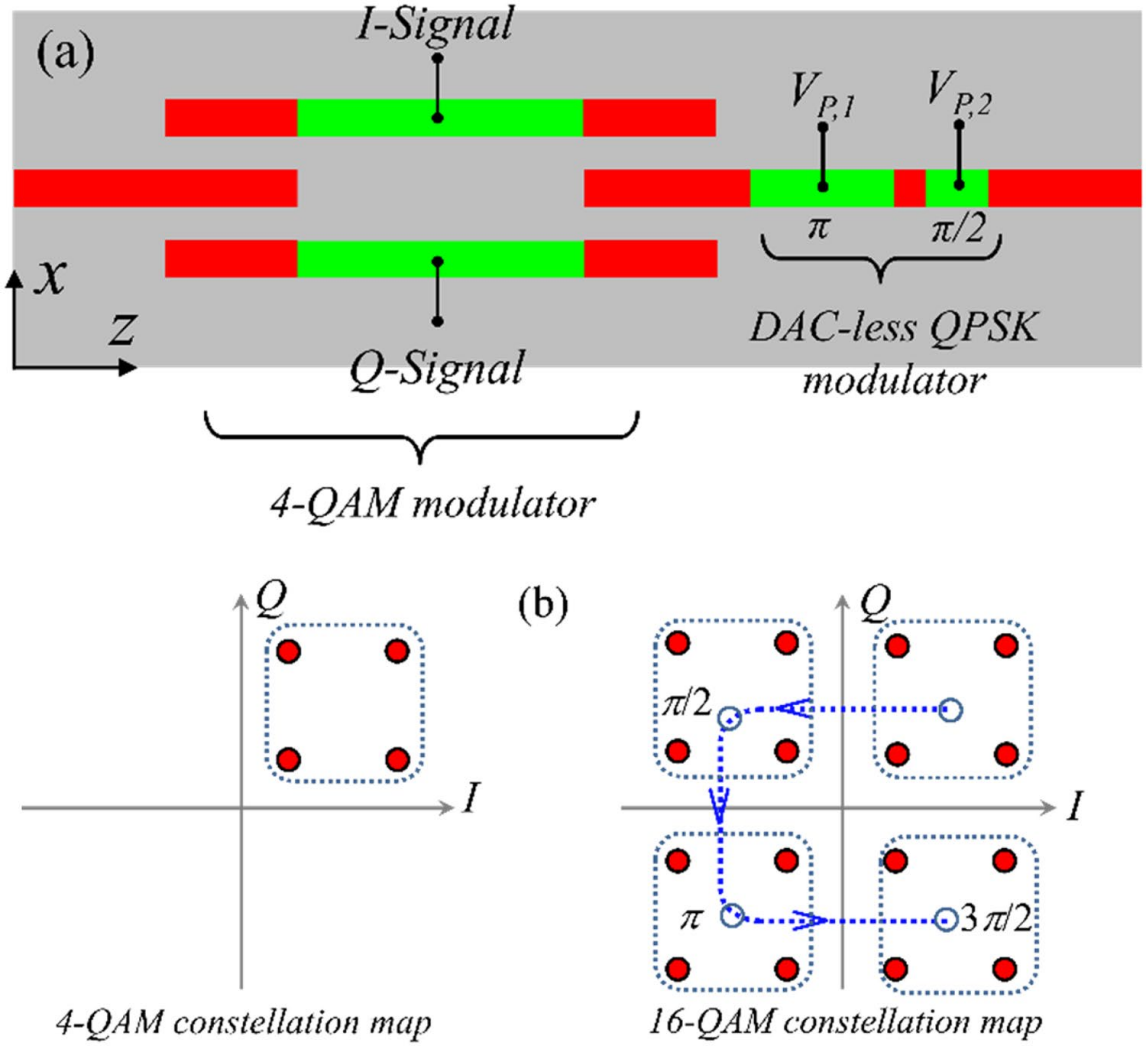
(**a**) A design for a 16-QAM modulator formed by cascading a 4-QAM and a QPSK modulator. (**b**) The constellation map of 16-QAM (right) which can be achieved by shifting the constellation map of 4-QAM (left) by *π*/2, *π*, and 3*π*/2.

As mentioned, the proposed structure can also be used to realize more complex modulation schemes such as 16-QAM. A schematic view of a possible design for a 16-QAM modulator based on the proposed 4-QAM is depicted in Fig. 9a. The 16-QAM is principally realized by introducing controlled phase shifts on the output of a 4-QAM modulator. This method was proposed in^57^ where an MZI structure had been used as the phase-shifting modulator (i.e. QPSK modulator). Here, instead of using an MZI as the QPSK element we have used the symmetry properties of the active waveguide to design a binary-weighted digital-to-analog-converter (DAC)-less QPSK modulator^20^. Considering the required phase shift of *π* and *π*/2 for the first and the second phase shifter (see Fig. 9a) the *on*- and *off*-state bias voltages of both active waveguide segments of the QPSK modulator are set to be *V*_*P,off*_ = 2.15 V and *V*_*P,on*_ = 4.21 V which are corresponding to the same propagation loss value of 1.76 dB/μm and effective index different of Δ*n*_*eff*_ = *n*_*eff,on*_ − *n*_*eff,off*_ = 0.2. The constellation map of the 4-QAM and 16-QAM modulators are presented in Fig. 9b. As can be seen, the constellation map of the 16-QAM modulator can be generated by introducing *π*/2, *π*, and 3*π*/2 phase shifts in the constellation map of the 4-QAM modulator. For this design, the IL and the energy consumption of the 16-QAM modulator are estimated to be 14 dB and 29fJ/bit, respectively. Using the sharp variations of the waveguide propagation loss to *V*_*S*_ (around the ENZ point), one can reduce the power consumption at the cost of a higher amount of IL. For example by choosing *V*_*P,off*_ = 2.8 V and *V*_*P,on*_ = 3.37 V the 16-QAM energy consumption will be ~ 2.5fJ/bit while its IL will be increased to 32 dB. Although this amount of loss is high in comparison to the 3.9 dB insertion loss of the proposed 4-QAM but is not too far from the IL of 16-QAM modulators proposed in^58^ and^55^.

A comparison between the performance parameters of the proposed 4-QAM and 16-QAM modulators and some of the previously reported QPSK and QAM modulators are provided in Table 3. As mentioned, ITO-based QAM modulators have not been reported previously and QAMs listed in this table are design in silicon-organic-hybrid (SOH) plasmonic waveguides^55,58^. According to this table, the proposed 4-QAM modulator is highly compact and therefore, its IL and power consumption is lower than the QPSK modulators of^10,20^. The 16-QAM modulator also is smaller than its SOH counterparts and its predicted bit rate is at least an order of magnitude larger than the others. The modulator power consumption is much lower than^55^ but is slightly higher than what reported for^58^. As mentioned, to reduce the power consumption and reach the lowest energy dissipation, one can sacrifice the IL which according to our estimation can result in energy consumption of 2.5fJ/bit at the expense of 32 dB insertion loss.

**Table 3:** Comparison of the properties of the proposed 4-QAM and 16-QAM modulator and some of the previously reported QAM and QPSK optical modulators.

Device type	Length (μm)	IL (dB)	Energy/bit (fJ)	Bit per symbol	Bit rate (Gbit/s)
ITO-QPSK^10^ ^a^	17.78	5.7	–	2	726
ITO-QPSK^20^	384.2/130.9	5.3/4.8	4000/1500	2	100
SOH-QAM^50^	1000	20^b^	18	4	52
SOH-QAM^55^	1500	30^b^	640	4	112
ITO-4-QAM (this work)	1.5	3.9	2	2	560
ITO-16-QAM (this work)	7.31/5.55	14/32	29/2.5	4	1120

^a^Energy consumption is not reported but the overall modulation system requires a DAC which substantially increases energy consumption.

^b^These references present measurement results.

## Discussion

An ITO-based active waveguide was proposed and its geometrical parameters were optimized to achieve widely tunable effective index and propagation loss variations for a range of applied bias voltages. The symmetry properties of the effective index and propagation loss of the waveguide around the ENZ bias point of the ITO were then used to design optical modulators in the MZI structure. A binary modulator and also a QAM modulator (without the use of any additional phase shift element) were proposed and simulated. For binary modulator, the IL, ER, and V_*π*_L_*π*_ FOM were found to be 1.24 dB, 54 dB, and 6.4 V.μm, respectively, and for the 4-QAM modulator the overall length, energy consumption, and bit rate, respectively, were estimated to be 6.2 μm, 2fJ/bit and 560Gbps. Then the symmetry properties of the proposed waveguide were further exploited to design a 16-QAM modulator by cascading the designed 4-QAM with a QPSK modulator formed by two active waveguide sections. The design of ITO-based QAM modulators was here reported for the first time and the abovementioned performance parameters show the unique properties of these modulators in terms of footprint, energy consumption and modulation-speed.

## Methods

**Figure 10:**
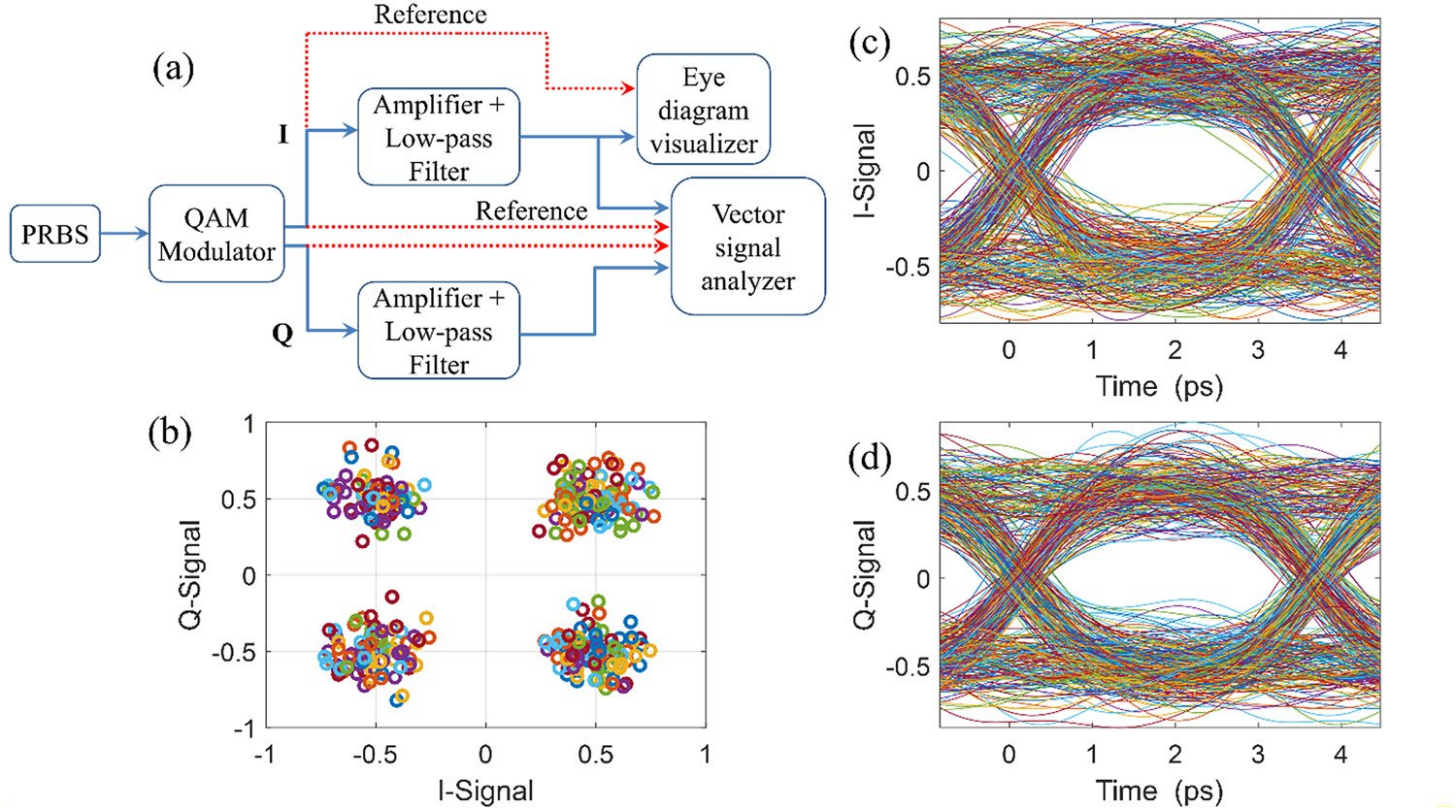
(**a**) Normalized constellation points of 4-QAM modulation system. The overall channel SNR and offset are estimated to 20 dB and ± 30%, respectively. (**b**) Eye diagram of *I-* and *Q-*Signals for a pseudo-random bit sequence (PRBS) with a frequency of 280 GHz.

To simulate the operation of the proposed structure as a QAM modulator we have used the INTERCONNECT module of the Lumerical software. The block diagram of the simulation setup is presented in Fig. 10a. The “PRBS” generates a pseudo-random bit sequence of 280Gbit/sec. The estimated IL and rise/fall times of the designed QAM4 modulator is used in “QAM Modulator” element and “Amplifier” and “Low-pass Filter” elements are included to simulate the practical situation^59,60^.

The constellation map is shown in Fig. 10b and eye-diagram for I- and Q-Signal are shown in Fig. 10c,d, respectively. As it is apparent, the constellation points of the modulation system are separated and our proposed device works properly. Also, both of the I- and Q-Signal eye diagrams are open in both amplitude and time directions for a bitstream of 280Gbit/sec.

**Figure 11:**
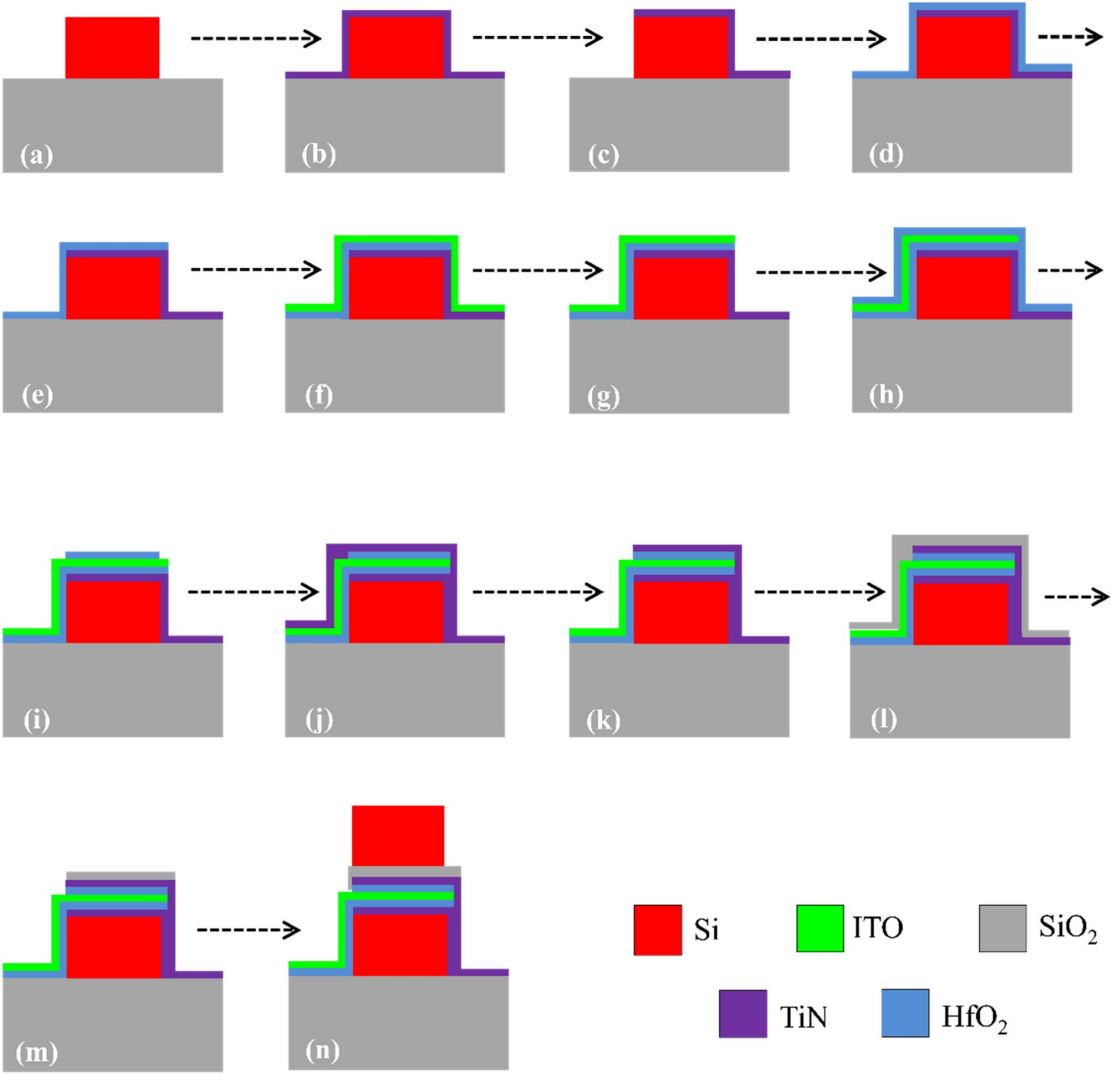
The suggested process flow for the fabrication of te proposed active waveguides.

Based on the previous experimental works a process flow for the fabrication of the proposed active waveguide is presented in Fig. 11. In the fabrication steps of the active waveguide, the definition of different patterns should be carried out using high-resolution lithography methods such as electron-beam-lithography (EBL) in combination with selective etching techniques such as plasma or deep reactive ion etching (DRIE)^13,14^. The TiN, HfO_2_ and ITO layers can be deposited through RF magnetron sputtering or atomic layer deposition (ALD), where the carrier concentration of deposited ITO can be controlled by tuning the oxygen concentration during deposition^14^. Moreover, the top silicon stripe can be formed by the epitaxial growth of silicon on top of the MOS structure or deposition of hydrogenated amorphous polysilicon via low temperature-PECVD. To reduce the impact of metal contacts on the optical mode, they should be placed a few microns away from the modulator. Starting from an SOI wafer the lower silicon stripe can be formed by EBL lithography and etching. The rest of the steps are presented in Fig. 11. Following different steps of Fig. 11a–l, the process fellow for fabrication of the proposed waveguide can be listed as (a) Starting with an SOI wafer and formation of the lower silicon stripe, (b) TiN deposition using ALD or RF Sputtering, (c) TiN patterning through EBL and Plasma or DRIE etching, (d) HfO_2_ deposition using ALD, (e) HfO_2_ patterning through EBL and Plasma or DRIE etching, (f) ITO deposition using ALD or RF Sputtering (doping level of ITO should be controlled through adjusting the oxygen concentration), (g) ITO patterning through EBL and Plasma or DRIE etching, (h) HfO_2_ deposition using ALD, (i) HfO_2_ patterning through EBL and Plasma or DRIE etching, (j) TiN deposition using ALD or RF Sputtering, (k) TiN patterning through EBL and Plasma or DRIE etching, (l) SiO2 deposition through sputtering, (m) SiO2 patterning through EBL and Plasma or DRIE etching, (o) Top silicon strip deposition using PECVD or epitaxial growth.

**Figure 12:**
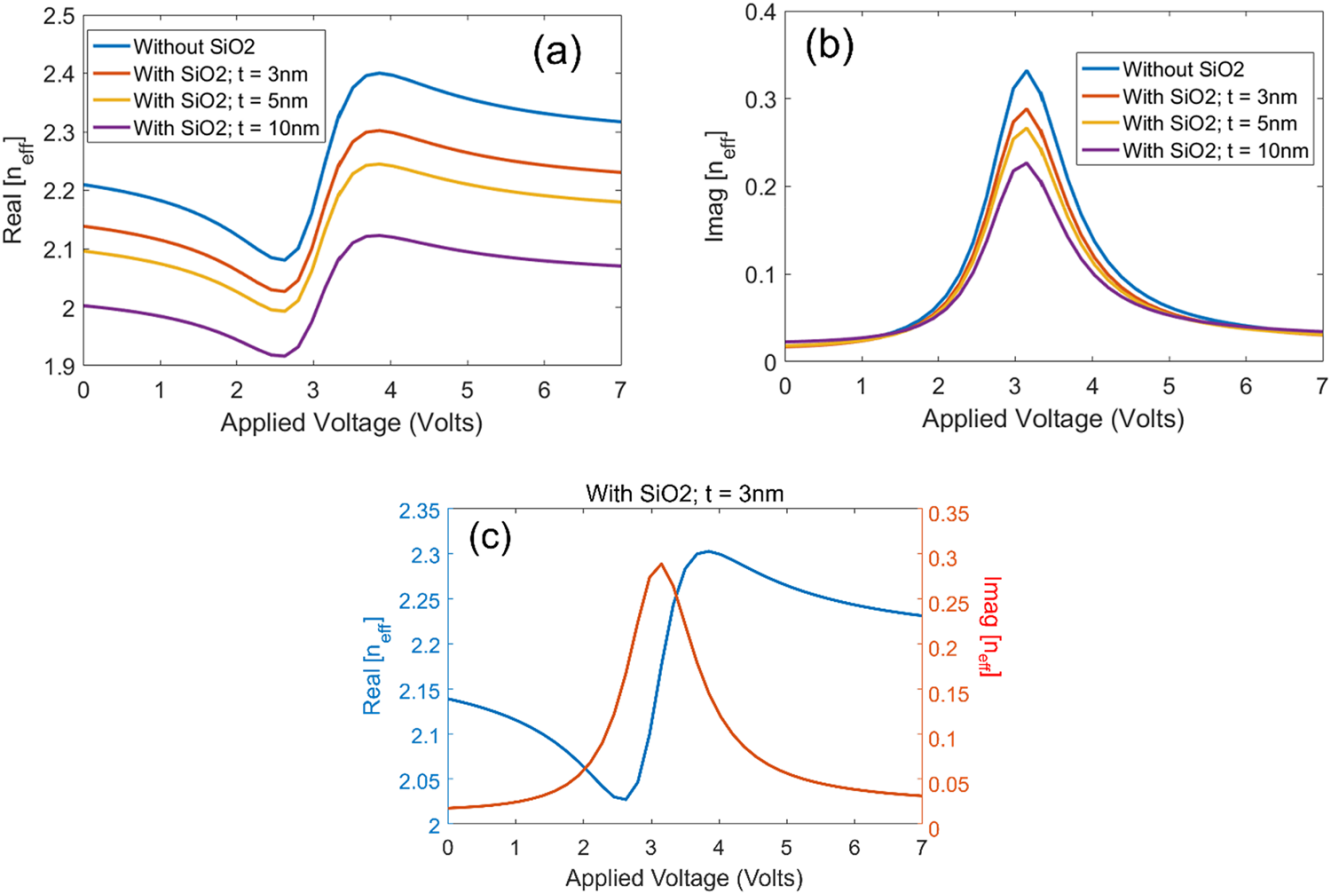
(**a**) Real and (**b**) Imaginary parts of the *n*_*eff*_ for different applied voltage values without SiO2 layer and with SiO2 thickness of 3 nm, 5 nm and 10 nm. (**c**) Real and imaginary parts of *n*_*eff*_ of the active waveguide with SiO2 thickness of 3 nm.

Abovementioned fabrication process results in an active waveguide with a small difference with the original waveguide of Fig. 1. Here a thin SiO2 layer is deposited between the upper TiN layer and silicon. The thin SiO2 film is used as sacrificial layer between TiN and silicon because successful grown of silicon on TiN has not been reported in the literature. To study the effect of SiO2 layer and find the proper thickness for this layer we have calculated the effective index for different values of applied voltage with SiO2 thickness of 3 nm and 5 nm and 10 nm. The real and imaginary parts of neff is plotted in part (a) and (b) of Fig. 12, respectively. As can be seen, presence of the SiO2 layer decreases the variations in the imaginary part of the *n*_*eff*_ in response to the applied voltage and shift the real part of the effective index to lower values.

In order to each maximum variations in real and imaginary parts of *n*_*eff*_ and minimize the effect of SiO2 layer on field profile we select the thickness of 3 nm for this layer. The real and imaginary parts of *n*_*eff*_ of the active waveguide with SiO2 thickness of 3 nm is plotted in Fig. 12c. Effective index variations have the same symmetry properties as the original waveguide and addition of the SiO2 layer does not change the modulator designs significantly. Since the effective index variations is a result of the ENZ in ITO the peak value of imaginary part of *n*_*eff*_ and maximum variations in real part of *n*_*eff*_ appears around the value of 3.1 V. Therefore, the bias voltages in design of different modulators can be kept the same as the original design. However, since the inclusion of a 3 nm SiO2 layer decreases peak value of the imaginary part of the *n*_*eff*_ by 15%, the active waveguide length in absorption modulator (Fig. 5) should be increased from 1.0 mm to 1.15 mm to reach the same results. Also, since the MOS structure is formed by the TiN/HfO2/ITO/HfO2/TiN stack, addition of the SiO2 does not change the capacitance of the structure and therefore, speed and power consumption calculations remain the same as the original designs.

## Data Availability

The calculated results during the current study are available from the corresponding author on reasonable request.
